# Tubastatin A inhibits HDAC and Sirtuin activity rather than being a HDAC6-specific inhibitor in mouse oocytes

**DOI:** 10.18632/aging.101867

**Published:** 2019-03-26

**Authors:** Yun-Jung Choi, Min-Hee Kang, Kwonho Hong, Jin-Hoi Kim

**Affiliations:** 1 Department of Stem Cell and Regenerative Biotechnology, Humanized Pig Research Center (SRC), Konkuk University, Seoul 143-701, Republic of Korea; * Equal contribution

**Keywords:** RNA-seq, germinal vesicle oocyte, meiotic oocyte maturation, HDAC6, sirtuin family, tubastatin A

## Abstract

Tubastatin A (TubA) is a highly selective histone deacetylase 6 (HDAC6) inhibitor. As expected, mouse germinal vesicle oocytes fail to extrude the first polar body following TubA treatment. However, a previous study demonstrated that homozygous *Hdac6* knockout (KO) mice can be viable and fertile. Therefore, we asked whether TubA is indeed a specific inhibitor of HDAC6 activity. RNA-sequencing and *in silico* analysis demonstrated that the TubA-treated group presented significant changes in the expression of *Hdac* subfamily genes such as *Hdac6*, *10*, and *11*, and *Sirtuin 2*, *5*, *6*, and *7*. Additionally, gene expression related to the p53, MAPK, Wnt, and Notch signaling pathways in the TubA-treated group were increased significantly; in contrast, gene expression related to metabolism, DNA replication, and oxidative phosphorylation was decreased significantly. Furthermore, gene expression related to cell cycle, cell structure, pyrimidine metabolism, pentose phosphate pathway, mitochondrial activation, proteasome pathway, RNA polymerase, DNA replication, cyclin-dependent kinase, nucleolar activity, and MI arrest were significantly decreased, indicating that TubA-induced abnormal meiotic maturation and oocyte senescence may be due to the combined effects of HDAC and Sirtuin inhibition, and not HDAC6 inhibition alone. Thus, we believed that this system could provide a model for monitoring the effects of TubA on mouse oocytes.

## Introduction

The balance between deposition and removal of histone modifications plays a critical role in the regulation of gene expression and maintenance of cellular homeostasis [1]. Epigenetic marking is known to be mediated by proteins that add, remove, or interpret the modified structures, referred to as “writers”, “erasers”, and “readers”, respectively [2]. Epigenetic changes controlled by the opposing activity of two classes of enzymes, histone acetyl transferases (HATs) and histone deacetylases (HDACs), can lead to altered phenotypes and gene expression [3]. Generally, HATs are categorized into two main families, type A and type B. Although little is known about type B HATs, type A have been characterized and can be divided into three major families: (1) GNAT family (PCAF and GCN5), (2) p300/CBP proteins, and (3) MYST proteins; meanwhile, HDACs constitute one superfamily and have been categorized into four main classes (class I, II, III, and IV). Among these, class III HDACs comprise seven Sirtuins (Sirt 1-7), unlike others, defined by their deacetylase activity that is dependent on nicotinamide adenine dinucleotide (NAD+) [4,5].

Various HDAC inhibitors (HDACis) are known to selectively alter gene transcription by promoting histone acetylation [6]. Moreover, they can increase the acetylation of many histone and nonhistone proteins, modifying their function or activity [7]. A good example of an HDACi is Tubastatin A (TubA), which drives cell cycle arrest, suggesting HDACs as therapeutic targets for cancer, chronic immune and inflammatory disorders, Alzheimer’s disease, and other tauopathies [8,9]. TubA is known as a potent and highly selective HDAC6 inhibitor with an IC50 of 15 nM and a greater than 1000-fold selectivity for all other isoforms, except HDAC8 (57-fold) [10]. HDAC6 is a unique member of the class IIb HDACs, possessing two catalytic domains and exhibiting a predominantly cytoplasmic localization [11]. It regulates various cellular processes including microtubule-based transport, cell motility, endocytosis, cell migration, autophagy, and aggresome formation by deacetylating non-histone proteins such as α-tubulin, cortactin, and heat shock protein 90 (HSP90) [12–16]. Inhibition of HDAC6 activity by TubA supplementation *in vitro* has been reported to lead to the hyperacetylation of tubulin and microtubules [12]. However, the molecular basis underlying the choice between abnormal oocyte maturation and acetylation homeostasis by TubA is not well understood.

Knockout (KO) mouse models provide a valuable experimental tool for the evaluation of gene function and there are various reports on the generation of *Hdac6* KO mice [13]. Although homozygous *Hdac6* KO mice exhibited global tubulin hyperacetylation, they were shown to be viable and fertile and presented no major observable phenotype [14]. Recently, however, we and others reported that TubA treatment induced cell cycle arrest with a failure of spindle migration and actin cap formation in mouse oocytes [15,16], indicating that most of the oocytes were arrested at an metaphase I (MI)- or a germinal vesicle breakdown (GVBD)-like stage. Taken together, these observations question the *in vivo* role of acetylated microtubules and the contribution of tubulin acetylases and deacetylases to cell function, and whether TubA inhibitory activity is indeed specific to HDAC6. To address these questions, TubA-supplemented oocytes were subjected to RNA-seq analysis to elucidate the underlying mechanism of TubA-induced abnormal oocyte maturation. In conclusion, RNA-sequencing and *in silico* pathway analysis demonstrated the potential for using mouse oocytes as an *in vitro* platform for the systematic validation of chemotherapeutic targets.

## RESULTS

To determine whether the wave of histone acetylation/deacetylation is of functional significance for the mouse oocyte meiotic resumption, mouse oocytes were allowed to undergo meiotic maturation with or without TubA, a potent inhibitor of HDAC6. Most control oocytes progressed to germinal vesicle breakdown (GVBD) at 4 h, first metaphase (MI) at 8 h, first anaphase and telophase (AT1) at 9.5 h, and second metaphase (MII) oocytes with first polar body at 12 h. However, oocytes exposed to 20 μM TubA [15] failed to transition through the GVBD to MI or AT1 stages.

### RNA-seq analysis of mouse oocytes

**Figure 1:**
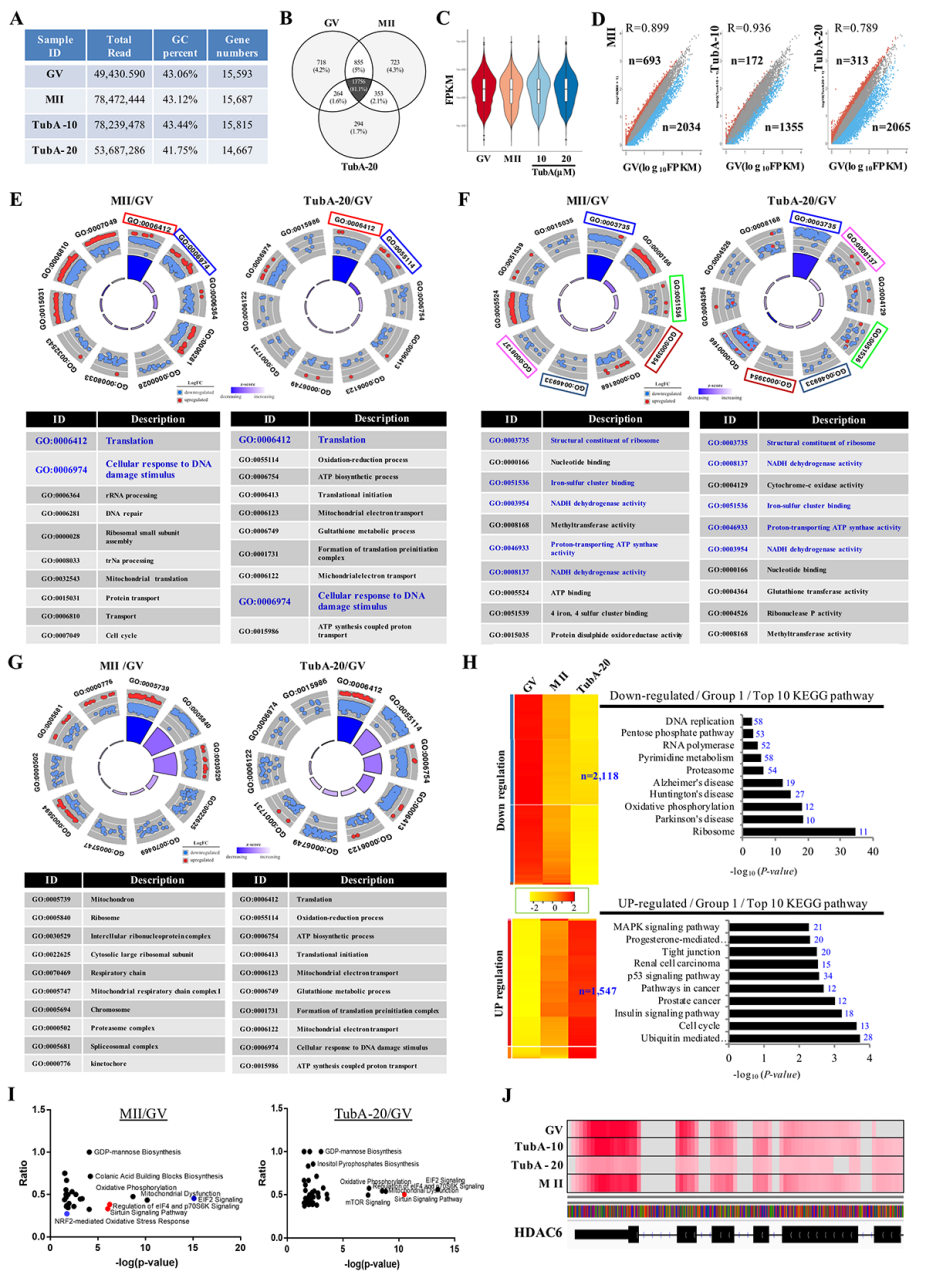
**Gene expression is altered in TubA treated oocytes.** (**A**) Sequence reads and number of unique genes in GV, MII, and the TubA (10 and 20 μM)-treated group. (**B**) Venn diagram showing the overlap between DEGs in GV, MII, TubA-10-, and TubA-20-treated oocytes. (**C**) Violin plots of differential gene expression profiles identified by SCPattern analysis. The *y*-axis indicates normalized expression values, FPKM. The *x*-axis indicates oocyte stage or TubA dosage of each sample. (**D**). Scatterplot comparing the transcriptomes of GV, MII, with or without TubA-10 and/or TubA-20. Blue dots indicate significantly decreased genes, while red dots indicate more highly expressed genes. (**E-G**). GO circle plot analysis of RNA-seq in MII/GV and TubA20/GV pairs. The outer circle shows a scatter plot for each term of the log_10_FC of the assigned genes in each enriched gene ontology (GO) term: biological process (**E**), molecular function (**F**), cellular component (**G**). Red circles display upregulation and blue circles display downregulation by default. The inner ring is a bar plot where the height of the bar indicates the significance of the GO terms (log_10_-adjusted p value), and color corresponds to the z-score: green, decreased; red, increased; and white, unchanged. (**H, I**) Canonical signaling pathways enriched in MT/GV and TubA-20/GV differentially expressed genes (ingenuity pathway analysis [IPA]). The ratio of differentially expressed genes in the pathways is shown for pathways with Benjamini-Hochberg-corrected p values <0.01. Activated or repressed pathways in (**I**) are shown as red and blue dots, respectively. Specific enriched pathways are highlighted. (**J**) Genome browser view depicting differential expression in fragment count for the *Hdac6* gene. At the gene level, overall, there is a large difference between control-GV (or –MII) and TubA-20, as shown in read coverage profiles. Red color indicates abundant transcripts of *Hdac6* mRNA.

Control GV- and MII-stage oocytes, as well as TubA-supplemented oocytes (10 and 20 μM) were subjected to RNA-seq to elucidate the underlying mechanism of abnormal oocyte maturation. As shown in Fig. 1A, a total of 49,430,590, 78,472,444, 78,239,478, and 53,687,286 reads were obtained from four RNA-seq libraries from GV, MII, TubA-10 (10 μM treatment) and TubA-20 (20 μM treatment), respectively. To reveal the molecular events underlying transcriptomic profiles, sequence reads were mapped to a reference transcriptome of control GV- or MII-stage oocytes. From the above reads, 15,593, 15,687, 15,815, and 14,667 genes from GV, MII, TubA-10, and TubA-20, respectively, were mapped to a unigene. To determine differences in gene expression between TubA-treated and control GV or MII oocytes, gene expression data from control GV oocytes were compared to data from control MII (MII/GV) or TubA-treated oocytes (TubA/GV). MII/GV and TubA/GV pairs had 13,756 commonly expressed genes, while 718, 723, and 294 differentially expressed genes (DEGs) were unique to GV, MII, and TubA, respectively (Fig. 1B). After normalization (Fig. 1C), 2,034 and 693 DEGs in MII/GV pairs were significantly up- and downregulated (R=0.899; log_10_FPKM, FC>2), respectively, whereas 2,065 and 313 DEGs were significantly up- and downregulated, respectively, in TubA20/GV pairs (Fig. 1D). All the DEGs with FC>2 and FDR<0.1 in one comparison were selected for canonical pathway analysis using IPA software. *P*-values were calculated based on the number of DEGs and total mapped genes, as described previously [17].

### Gene network and IPA analysis

An RNA-seq-based transcriptome analysis was used to investigate key genes involved in abnormal meiosis of mouse oocyte maturation. The most enriched gene ontology (GO) terms for biological process, cellular component, and molecular function using GOplot analysis are shown in Fig. 1E-G, respectively. The common GO terms from MTII/GV and TubA/GV pairs are translation and cellular response to DNA damage stimulus for biological process (Fig. 1E), structural constituent of ribosome, iron-sulfur cluster binding, proton-transporting ATP-synthase activity, and NADH dehydrogenase activity for cellular component (Fig. 1F). However, no common category was found for molecular function (Fig. 1G). Next, we examined the dysregulation of canonical signaling pathways using Ingenuity Pathway Analysis (IPA) and plotted the ratio of dysregulated genes in specific pathways against their *p* value for overrepresentation (Fig. 1H and I). Of note, MII/GV pairs showed a negative feedback loop in EIF2 signaling and the NRF2-mediated oxidative stress response pathway (Fig. 1I, blue color).

An important quality parameter of RNA-seq mapping is the percentage of mapped reads, which is an indicator of the overall RNA-seq accuracy. When reads were mapped against the transcriptome of the *Hdac6* gene, the TubA-20 treated group showed marginally lower multi-mapping read percentages compared to control GV or MII oocytes (Fig. 1J), indicating that TubA is a critical inhibitor of HDAC6 and that our approach was shown to work well with normalized RNA-seq data.

**Figure 2:**
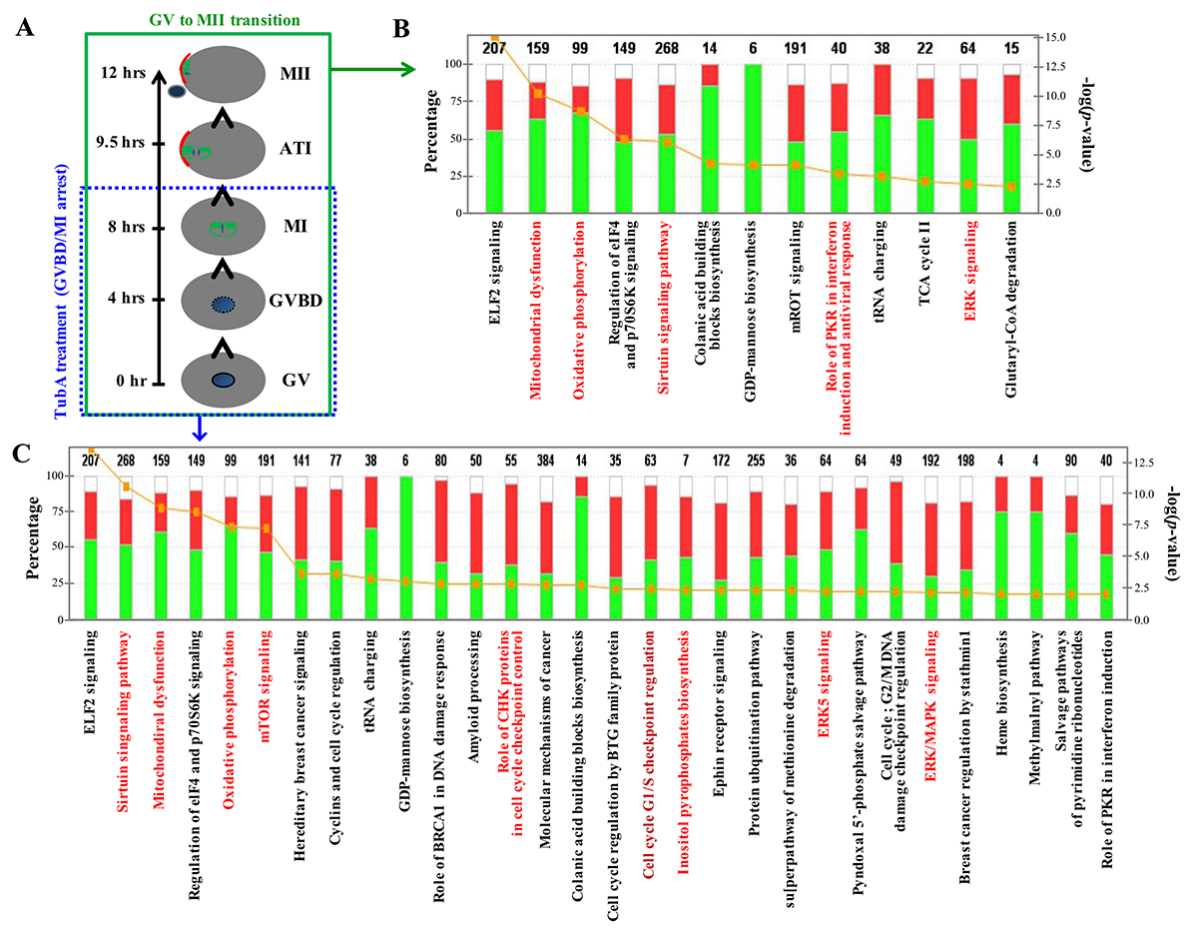
**Major canonical pathways influenced TubA in control MII/GV and TubA/GV pairs.** (**A**) Time schedule of control GV to MII oocyte transition. (**B, C**) Ingenuity pathway analysis (IPA) and functional categorization of the top selected genes by RNA-Seq of control MII/GV (B) and TubA/GV (C) pairs. The secondary Y-axis shows the -log (p-value) of the probability for genes in a data set to associate with identified pathways by chance. A threshold p value of 0.05 is presented as a yellow dotted line. The ratio of the number of genes from the data set that map to a given pathway divided by the total number of genes that map to the canonical pathway is shown as a solid line.

After RNA-seq quantification of the normalized data, we examined DEG profiles between control (GV or MII) and TubA-treated groups. The number of up- and downregulated DEGs in the top 10 enriched pathways are shown in Supplementary Table 1 and 2. Of note, metabolic-related pathways (pentose phosphate, pyrimidine metabolism, oxidative phosphorylation, and ribosome pathways) in the TubA-treated group were strongly downregulated. However, the top 10 upregulated KEGG pathways in the TubA-treated group were MAPK signaling, progesterone-mediated oocyte maturation, p53 signaling, insulin signaling, and cell cycle (Supplementary Table 1). As shown in Fig. 2B and C, IPA canonical pathway analysis showed that EIF2 signaling, mitochondrial dysfunction, oxidative phosphorylation, regulation of EIF4 and p70S6K signaling, Sirtuin signaling, colanic acid building block biosynthesis, GDP-mannose biosynthesis, mTOR signaling, role of PKR in interferon, induction and antiviral response, tRNA charging, and ERK signaling were simultaneously affected in both the MII/GV and TubA/GV pairs. However, a significant proportion of the genes in TubA-treated oocytes were enriched in cell cycle-related pathways (cyclins and cell cycle regulation, role of BRCA1 in DNA damage response, CHK proteins in cell cycle checkpoint control, cell cycle regulation by BTG family proteins, cell cycle G1/S checkpoint regulation, G2/M DNA damage checkpoint regulation, and MAPK/ERK signaling). The observed dysregulation of these genes would be expected to prolong cell cycle checkpoint signaling leading to a further delay or block in entry into meiosis.

### TubA effect is not solely mediated by HDAC6-specific inhibition in mouse oocytes

**Figure 3:**
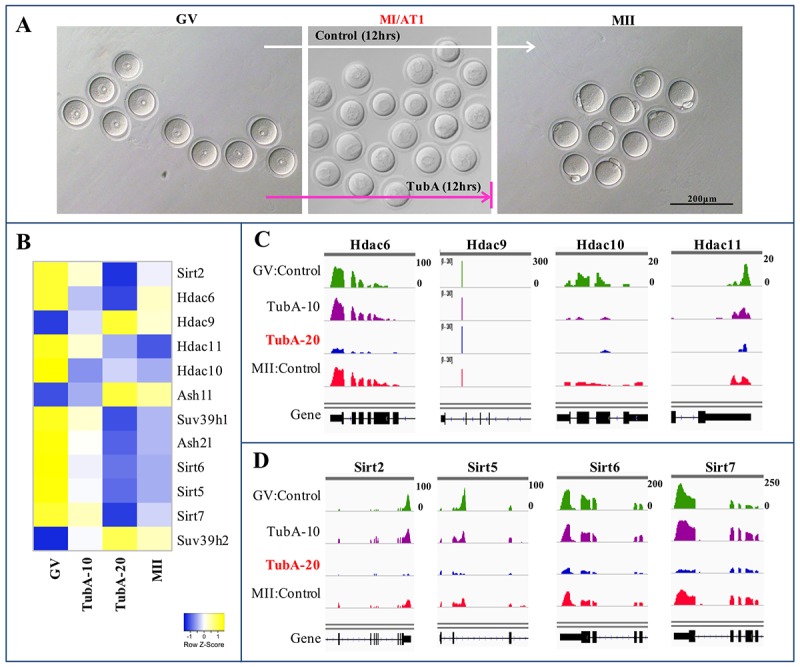
**Heatmap and IGV of DEGs in control GV, TubA (10 and 20 µM)-treated, and MII oocytes.** (**A**) A representative oocyte of control-GV, TubA-treated (10 and 20 μM), and control-MII-stage oocytes. Of note, control mouse GV oocytes can undergo meiotic maturation *in vitro*, whereas 20 μM of TubA supplementation inhibited progression through the MI (or AT1) to MII transition in mouse oocytes. (**C, D**) Visualization of mRNA expression enrichment of the Class IIb *Hdac* and Class III sirtuin loci using IGV.

TubA-treated oocytes underwent GBVD on schedule, but the meiosis I to meiosis II transition was impaired (Fig. 3A). Given that TubA is known as a specific inhibitor of HDAC6 [6], we examined whether inhibition or blocking the GV to GVBD or MI (or ATI) transition observed with TubA treatment resulted from disruption of *Hdac6*-specific gene expression. As shown in Fig. 3B and C, the expression of *Hdac6* in control MII-stage oocytes was marginally lower compared to control GV-stage oocytes, whereas *Hdac6* mRNA levels in TubA-treated groups were significantly decreased compared to control GV- or MII-stage oocytes. Similar results were obtained for *Hdac10* and *11* mRNA expressions, although the expression of these genes during the GV to MII transition in the control group was marginally higher than the TubA-treated group. Although *Hdac9* mRNA expression during the GV to MII transition showed a small decrease in the control group, expression in the TubA-treated group increased slightly or was not altered compared to the control during the GV to MII transition. Therefore, our RNA-seq analysis indicates that significant changes in the expression of these mRNAs, as well as the observed abnormal oocyte maturation, were closely related to TubA treatment.

Of the pathways analyzed, the Sirtuin signaling pathway exhibited the highest *p* value (*p* = 2×10^−17^), and is predicted to be activated in the TubA‐treated samples (Fig. 2C). Consequently, we examined Sirtuin family-related gene expression and found that TubA was not selective for HDAC6 alone, instead inhibiting both Class II (*Hdac6* and *9*) and III HDACs (*Sirt2, 5, 6*, and *7*) (Fig. 3B-D). Previous data suggested that a specific Sirt2 inhibitor blocked the progression of GVBD during *in vitro* oocyte maturation [18] and that *Sirt2* knockdown resulted in abnormal spindle and chromosome alignment during meiosis [19]. Furthermore, *Sirt6* or *7* deficiencies lead to replication stress, an important source of endogenous DNA damage [20,21]. Therefore, TubA-induced spindle or chromosome organization failure may be due to reduced *Sirt2*, *5*, *6*, and *7* mRNA expressions.

### Effects of TubA on the expression of oocyte maturation-related genes

**Figure 4:**
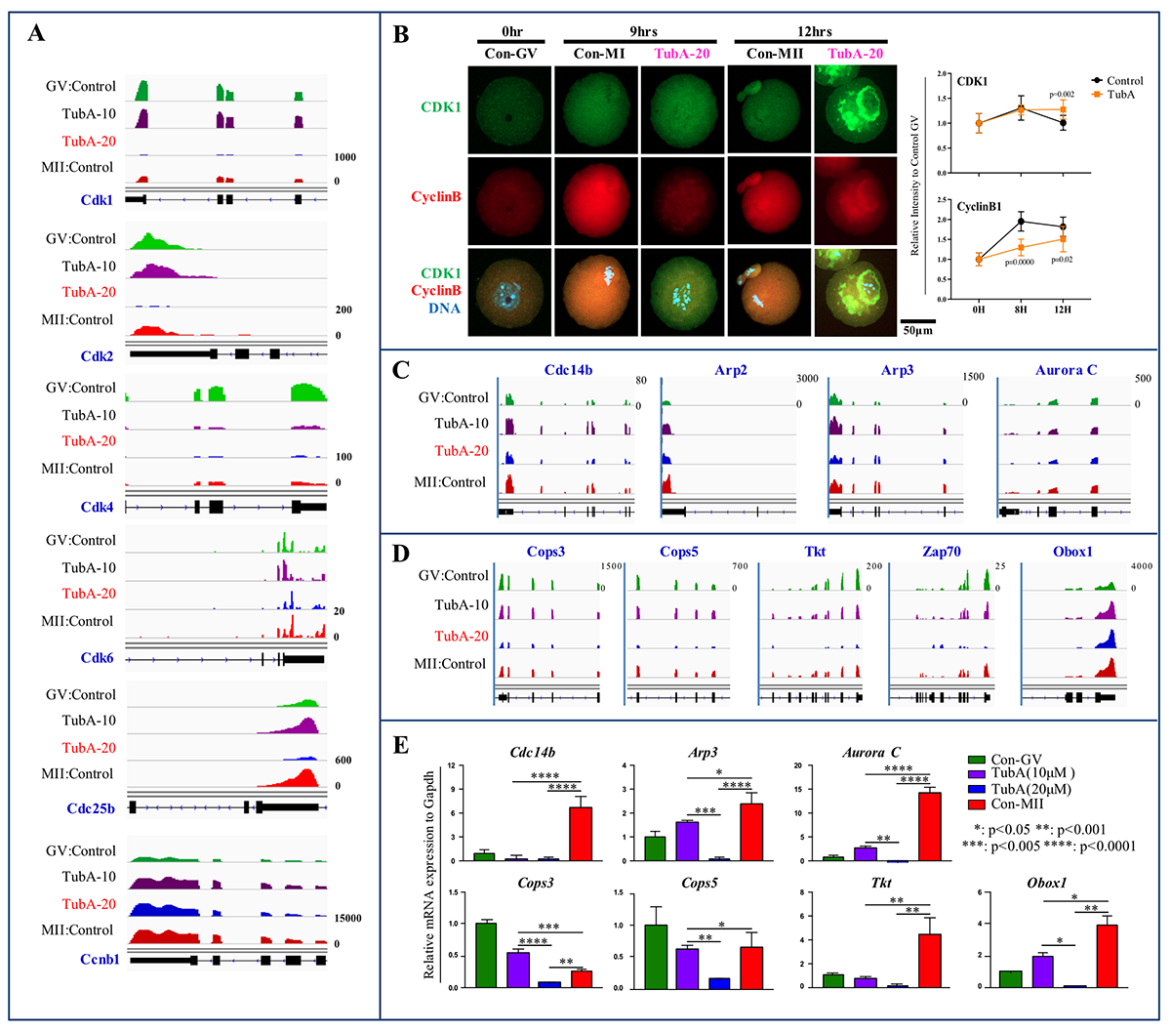
**Mechanistic target of maturation promoting factor (MPF) signaling is dysregulated in TubA-treated oocytes.** (**A**) Cyclin-dependent kinase (*Cdk1*, *2*, *4*, *6, Cdc25b*, and *Ccnb1*) mRNA expression profiles using IGV. (**B**) Confocal immunofluorescence analysis of CDK1 (green) and Cyclin B (red) in oocytes with/without TubA exposure during *in vitro* maturation at 0 h, 9 h, and 12 h. The experiment was repeated three times. DNA was stained with DAPI (blue). Right indicates quantification of each staining density. (**C, D**) mRNA expression profiles of cytokinesis- and MI arrest-related factors in mouse oocyte profiles in matched pairs of TubA (10 or 20 μM)-treated (TubA/GV pairs) versus untreated control groups (MII/GV pairs). (**E**) Confirmation of DEGs obtained from RNA-seq. Bar graph showing relative gene expression obtained from qRT-PCR analysis of selected genes. *, **, ***, and **** represent genes displaying statistically significant changes in expressions.

To examine cyclin-dependent kinase gene expression (*Cdk1, Cdk2, Cdk4,* and *Cdk6*), we visualized the enrichment of each mRNA expression at the candidate gene loci using Integrative Genomics Viewer (IGV). As shown in Fig. 4A, expression of these mRNAs in TubA-treated oocytes was significantly reduced compared to the control GV- or MII-stage oocytes. Even though mRNA expression levels of *Cdk1, Cdk2, Cdk4, Cdk6,* and *Cdc25b* in the TubA-treated groups showed a significant decrease, CDK1 and Cyclin B protein content in the TubA-treated groups increased continuously in a time-dependent manner (Fig. 4B) compared to the control. Previously, it was reported that protein synthesis rates were significantly reduced during oocyte maturation [22], indicating that the failure of developing oocytes in transition from the GV to MI or MI to MII stage following TubA treatment may be correlated with impaired CDK and Cyclin B accumulation; this may disrupt the nuclear shape and chromatin organization and subsequently lead to DNA damage accumulation and senescence. Furthermore, 19 genes involved in degradation through the 26S proteasome pathway (*Psmb5*, *Psmf1*, *Psmb4*, *Psmd14*, *Psmb7*, *Psmc5*, *Psmd13*, *Psmb6*, *Psma6*, *Psmc4*, *Psmb1*, *Psme2*, *Psmc3*, *Psmc2*, *Psmc1*, *Psma3*, *Psmd4*, *Psmd6*, and *Psmd8*), showed significantly decreased expression levels in the TubA/GV compared to the MII/GV pair (Supplementary Table 2). This may also explain why protein levels increased despite the downregulation of mRNA expression after TubA supplementation.

We next determined the effects of TubA on the expression of factors involved in the GVBD to MI transition (or MI to AT1 transition) arrest. As shown in Fig. 4C and D, the expression of MI arrest-related genes (*Cops3*, *Cops5*, *Tkt*, *Zap70*, and *Obox5*) and cytokinesis-related gene (*Arp2*, *Arp3*, *Aurora C*, and *Cdc14b*) in TubA-treated oocytes was significantly reduced compared to the control. Considering a relative increase in these transcript levels was observed during the GV to MII transition in control oocytes, a significant reduction in the levels of these transcripts in the TubA-treated group may result in the failure of spindle or chromosome organization during oocyte maturation. Additionally, there was a significant decrease in RNA polymerase (*Popr3h*, *Polr2e*, *Polr3k*, *Polr1e*, *Polr2l*, *Polr1d*, *Polr2k*, *Polr2i*, *Znrd1*, *Polr3a*, *Polr1c*, and *Polr3c*)- and DNA replication (*Rpa2*, *Pold4*, *Rfc3*, *Rfc4*, *Ssbp1*, *Rfc2*, *Pold2*, *Rnaseh2a*, *Fen1*, *Rnaseh2c*, and *Mcm5*)-related genes in the TubA/GV pair. Furthermore, gene expression related to cell structure (*Tubb1a*, *Tubb2b*, and *Tubb6*), mitochondrial activation (*Atp5D*, *Atp5E*, *Atp5h*, *Atp5k*, *Atp5j*, *Atp5O*, *Atp6v0d1*, *Ndufs6*, *Ndufs5*, *Ndufs8*, *Ndufs3*, and *Ndufs2*), pyrimidine metabolism (*Dtymk*, *Upp1*, *Znrd1*, *Tk2*, *Tyms*, *Nt5m*, *Nt5c3*, *Uck2*, *Entpd1*, *Nudt2*, *Nme6*, *Umps*, *Nme3,* and *Nme1*), and the pentose phosphate pathway (*Aldoa*, *Pgm2*, *Pgls*, *G6pdx*, *Fbp1*, *Pfkp*, *Dera*, *Tkt*, *Fbp2*, and *Gpi1*) also exhibited a significant decrease (Supplementary Table 2). Together, these observations suggest that TubA-treated oocytes may arrest at the MI stage due to reduced metabolism induced by the TubA treatment.

For verification of the RNA-seq data, the expression levels of these RNAs were quantified using quantitative real time reverse transcription PCR (RT-qPCR). For these experiments, a separate group of TubA-treated or control GV- or MII-stage oocytes was prepared under the same experimental conditions used for the RNA-seq analysis. For validation, we examined the expression of MI arrest-related genes (*Cops3*, *Cops4*, *Tkt*, *Zsp70*, and *Obox1*) and cytokinesis-related genes (*Cdc14b*, *Arp2*, *Arp3*, and *Aurora C*). As shown in Fig. 4E, we found that the expression of the selected mRNAs detected by RT-qPCR agree with the RNA-seq data.

### Effects of TubA on nucleolar activity

**Figure 5:**
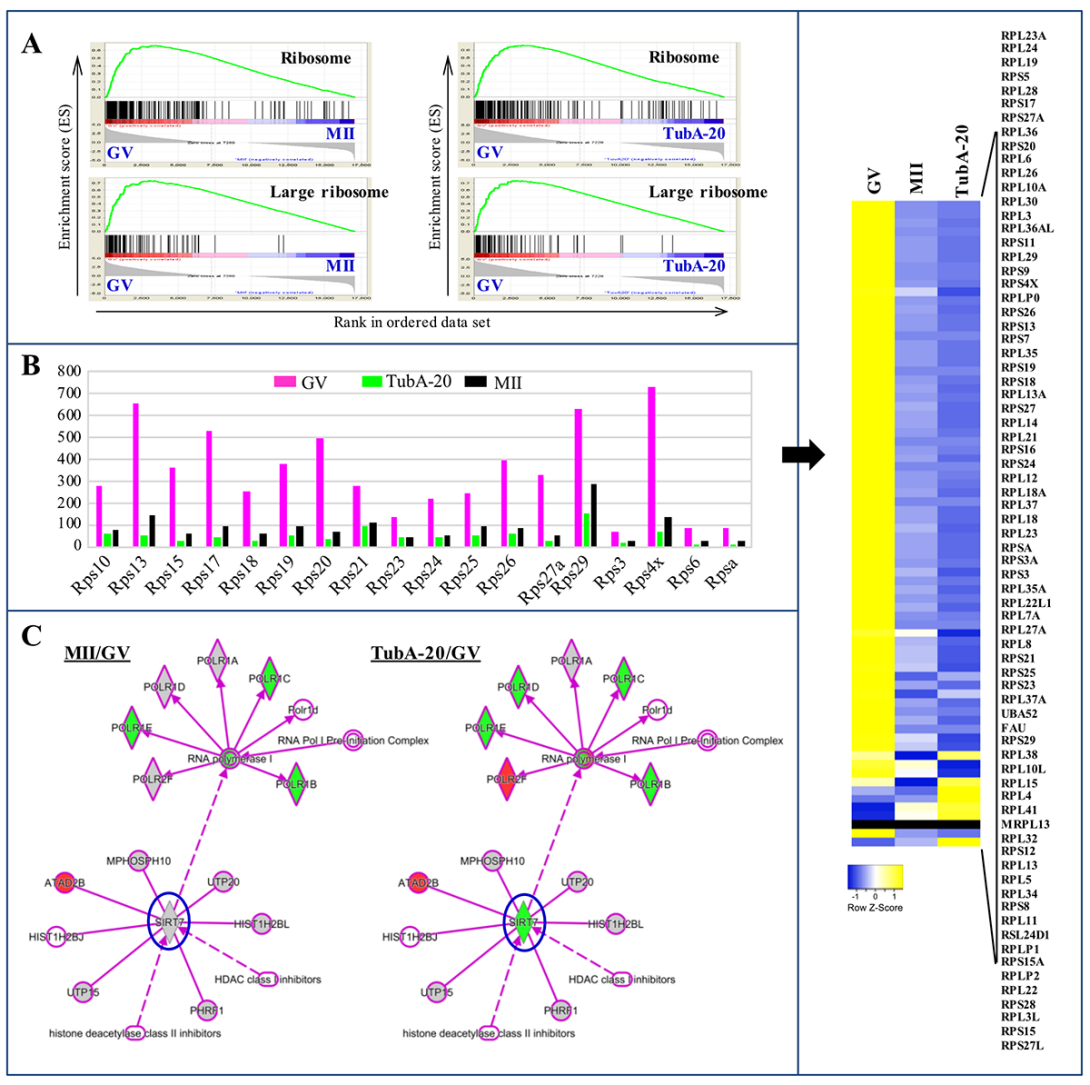
**Ribosome biosynthesis in GV, MII, and TubA-treated oocytes.** (**A**) GSEA plots showing preferential downregulation of the KEGG ribosome in matched pairs of TubA20 treated (TubA/GV pairs) versus untreated control (MII/GV pairs). (**B**) Expression of ribosome biosynthesis-related functional genes in GV-, TubA-20 treated, and MII-stage oocytes. Y-axis shows the FPKM values of genes inferred from the transcriptome data. Right indicates a heatmap showing DEGs of *Rps* family genes in GV, MII, and TubA-treated oocytes. (**C**) IPA analysis of MII/GV and TubA/GV pairs. Shapes and lines are color-coded based on predicted associations and functions. Green and red indicate up- and downregulated gene expression.

**Figure 6:**
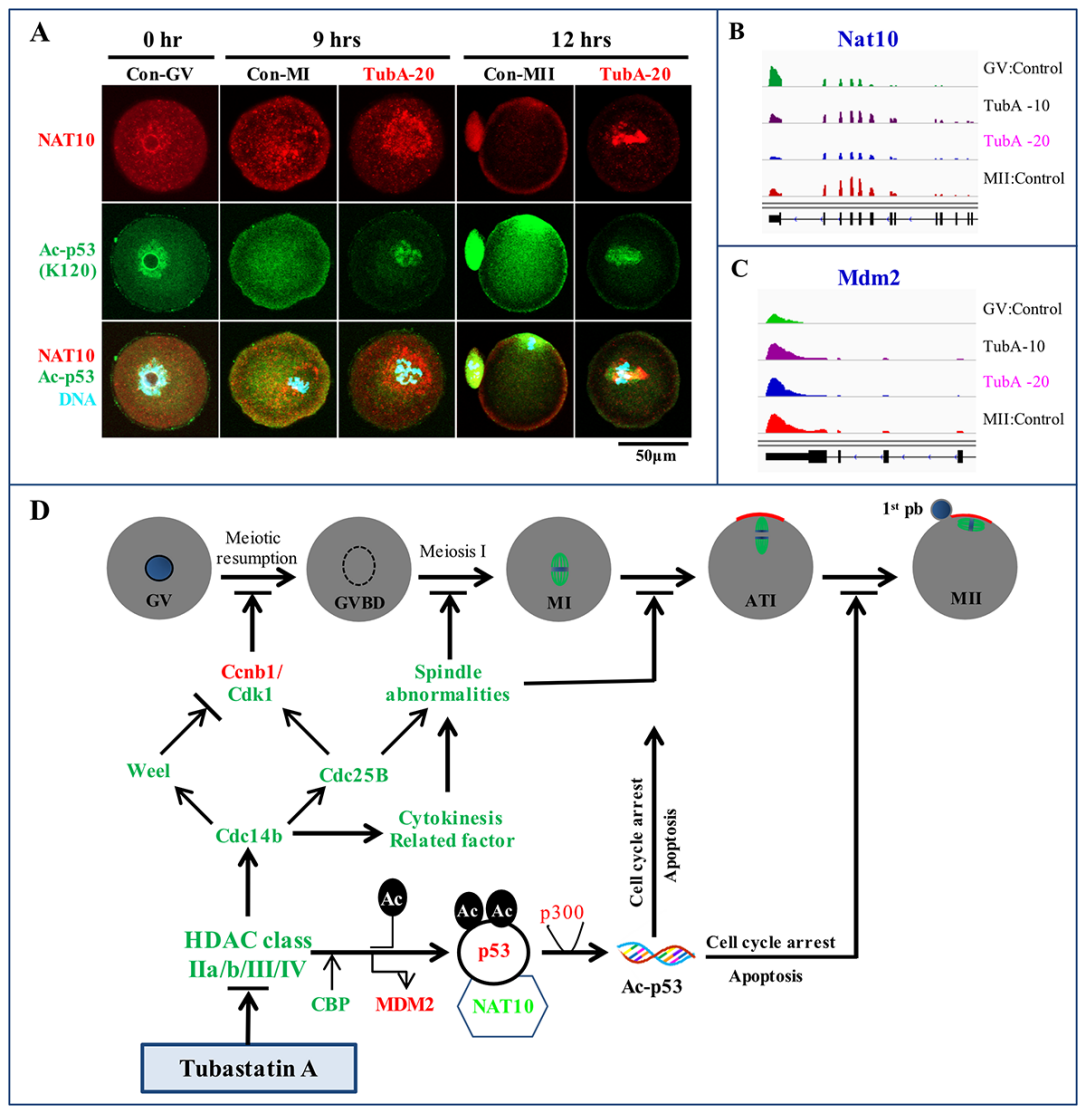
**Immunofluorescence analysis and a putative TubA mechanism in mouse oocytes.** (**A**) Confocal immunofluorescence analysis of NAT10 and Ac-p53 expression at K120. The experiment was repeated three times. (**B, C**) Visualization of *Nat10* and *Mdm2* mRNA expression profiles using the IGV. (**D**) An underlying mechanism to explain TubA roles during oocyte maturation in the mouse. Green or red colors indicate mRNAs and proteins expression down- or up-regulated by TubA treatment, respectively.

The nuclear structures responsible for ribosome biogenesis are nucleoli. To examine the roles of TubA in ribosome biogenesis, we performed an analysis of gene set enrichment using KEGG pathway mapping. The “ribosome” pathway was the top differentially expressed pathway with both the control (MII/GV) and TubA/GV pairs (Fig. 5A), with genes encoding ribosomal subunits such as *Rps5*, *6*, *10*, *13*, *15*, *17*, *18*, *19,* and *20* presenting significant reductions (Fig. 5B, left). Of the 73 transcripts that encode cytoplasmic ribosomal proteins from both the large and small ribosomal subunits, most transcripts in the control MII-stage oocytes showed a significant decrease compared to control GV oocytes. Notably, TubA-treated groups showed a greater reduction in mRNAs encoding these ribosomal proteins, with a few exceptions (Right, Fig. 5B). As shown in Supplementary Table 2, P*olr1e*, *Polr1d*, and *Polr1c* mRNA expression in the TubA/GV pair was significantly downregulated compared to the MII/GV pair. Furthermore, *Rpl3*, *Rpl5*, *Rpl11*, and *Rpl23* gene expressions as well as *p53*, *Mdm2*, *E2fs*, and *c-myc* in TubA/GV pair are significantly altered, compared to MII/GV pair (Fig.5B and Fig.6). Considering that initiation of RNA synthesis is associated with a characteristic remodeling of the nucleolar architecture, TubA may disrupt remodeling of the nucleolar architecture during oocyte maturation. As a result, perturbation of ribosome biogenesis in developing oocytes by TubA treatment may cause nucleolar stress, leading to cell cycle arrest in a p53-dependent manner.

Previous studies reported that SIRT7 was the only sirtuin localized to nucleoli [23,24] and that SIRT7 depletion led to hyperacetylation of H3K18 in the promoters of the *Rps20*, *Rps7*, *Rps14*, *Nme1*, and *Cops2* genes [25]. In this study, we found that *Cops3*, *Cops5*, and *Sirt7* gene expression was significantly downregulated (Fig. 3D and Fig. 4D). Considering that Sirt7 may be a positive regulator of rDNA transcription via its association with RNA Polymerase I [26], we propose that reduced rDNA transcription in TubA-stimulated oocytes may be associated with Sirt7 and linked to replicative senescence, which may be responsible for inducing oocyte aging (Fig. 5C).

### Effects of TubA on the regulation of NAT10, a nucleolar protein

In control oocytes, NAT10 protein expression patterns was changed from the nucleolus in GV oocytes to the cytoplasm in MI oocytes, and the membrane and first polar body of MII-stage oocytes (Fig. 6A). Nine hours after TubA supplementation, however, NAT10 protein staining was strongly localized to the peri-nucleus including the cytoplasm. After 12 h, NAT10 protein staining became limited to the peri-chromosomes. Of note, Ac-p53 (K120) staining patterns in both the control and TubA-treated groups were very closely correlated with those of NAT10. In addition, *Nat10* mRNA expression in the TubA-treated groups decreased slightly, whereas *Mdm2* mRNA expression was similar to control MII-stage oocytes (Fig. 6B and C). Collectively, immunostaining patterns in the TubA-treated groups demonstrate that nuclear NAT10 and Ac-p53 (K120) were correlated with the poorest outcome of meiotic exit compared to cytoplasmic and membrane-associated NAT10 and Ac-p53 (K120), indicating that the subcellular distribution may be important for the progression of meiotic oocyte maturation.

In conclusion, RNA-seq and *in silico* pathway analysis demonstrated the potential of using mouse oocytes as an *in vitro* platform for the systematic validation of chemotherapeutic targets. In this study, we propose a key underlying mechanism to explain the failure of meiosis in TubA-treated oocytes (Fig. 6D). These observations strongly suggest that TubA is not a specific inhibitor of HDAC6 in mouse oocytes. Rather, we believe that the effects of TubA disrupt spindle migration and asymmetric division in mouse oocytes, and then induce oocyte senescence and aging, which eventually results in female infertility.

## DISCUSSION

*In vitro* oocyte maturation in the presence of 20 μM TubA (p<0.05) is arrested at the MI or ATI stage, and shows abnormal spindle and chromosome alignment [15]. Here, we performed single oocyte RNA-Seq and analyzed the *in silico* data to address several unanswered questions regarding TubA effects and to determine the epigenetic effects of TubA on oocyte maturation and viability. Additionally, we used the R package GOplot, KEGG, and IPA analysis to identify the underlying mechanism of TubA-induced abnormal oocyte maturation.

TubA supplementation in culture medium resulted in the combined inhibition of *Hdacs* (*6*, *9, 10*, and *11*) and *Sirtuins* (*Sirt2, 5, 6*, and *7*) in cultured oocytes. TubA is a highly selective HDAC6 inhibitor and is reported to inhibit the enzymatic activity of HDAC6 in cells. In this and a previous study, the protein and mRNA levels of *Hdac6* in TubA-treated mouse oocytes were significantly reduced. This posed the question as to how a specific enzymatic inhibitor could result in mRNA degradation and reduction in protein expression. There are some possible mechanisms to explain how specific inhibition of an enzyme could result in mRNA degradation and subsequent reduction in protein expression. First, HDAC6 may directly target the *Hdac6* gene in a negative feedback mechanism, as described previously [27]. A second possible reason may be that the observed TubA effects were not specific to HDAC6. In this study, we found that both *Hdacs* (*6*, *9*, *10*, and *11*) and *Sirtuins* (*2, 5, 6,* and *7*) were dysregulated in the TubA-treated group compared to the control. Therefore, we believe that inhibition of both HDACs and sirtuins by TubA may disrupt spindle migration and asymmetric division in mouse oocytes. Furthermore, previous studies have demonstrated that histones are globally deacetylated during meiosis at the MI and MII stages by histone deacetylase activity in mammalian oocytes [28–31]. Taken together, our analysis suggest that TubA induced inadequate histone deacetylation causes chromatin perturbation, spindle defects, and chromosome misalignment [32].

Both of Sirt2 and Hdac6 are tubulin deacetylases and that are thought to function as essential coenzymes [33,34], and it has been shown that knockdown of either *Sirt*2 or *Hdac6* by siRNA disturbed the mouse meiotic apparatus [35]. In this study, the mRNA levels of both *Sirt2* and *Hdac6* were significantly downregulated in TubA-exposed oocytes (Fig. 3). Our results are consistent with previous data showing that a specific Sirt2 inhibitor blocked the progression of GVBD during oocyte maturation *in vitro* [18] and that *Sirt2* knockdown resulted in abnormal spindle and chromosome alignment during meiosis [19]. However, it remains to be determined how TubA treatment disrupts spindle assembly and chromosome movement during oocyte meiosis. Previous studies indicated that histone H4K16 and α-tubulin-K40 may be the potential targets of Sirt2 in modulating chromatin conformation and microtubule stability [33,36]. Therefore, we conclude that TubA supplementation induced the reduction of *Sirt2* mRNA expression, which in turn led to α-tubulin hyperacetylation and consequently contributed to the failure of spindle or chromosome organization or to aneuploidy.

We also observed a significant downregulation of *Sirt6* and *7* mRNA levels in TubA-treated oocytes, two genes that play a pivotal role in controlling meiotic progression and transcriptional regulation of ribosomal DNA, respectively [32,37,38]. *Sirt1-7* genes have all been knocked out in mice [21,39–48]. Among these, *Sirt6* KO mice died within one month of birth with accelerated aging, while *Sirt7* KO mice also died at a late stage of embryonic development. In somatic cells, SIRT7 depletion resulted in an increased mutation rate, sensitivity to different DNA damage agents, and abnormal rates of apoptosis. *Sirt6* or *7* deficiencies, similar to that observed in *Sirt2* KO cells [13,49], resulted in replication stress, which is an important source of endogenous DNA damage [20,21,45,50]. In addition, knockdown of *Sirt7* led to reduced RNA Pol I levels, but *RNA Pol I* mRNA levels was not changed. This suggests that Sirt7 plays a crucial role in connecting the function of chromatin remodeling complexes to RNA Pol I machinery during transcription [51,52]. Furthermore, decreased levels of *Sirt7* during senescence led to increased acetylated forms of NPM1, known to promote cellular senescence [53]. Thus, these observations suggest that the expression of both of *Sirt6* and *7* is critical for the meiotic maturation of mouse oocytes.

In this study, mRNA expression of most of cyclin-dependent kinases such as *Cdk1, Cdk2, Cdk4, Cdk6* and *Cdc25b* exhibited significant decreases in the TubA-treated group, whereas *Ccnb1* mRNA expression was increased slightly, or was similar, compared to control GV- or MII-stage oocytes. Previous studies demonstrated that *Cdk2*, *Cdk3*, *Cdk4 or Cdk6* KO mice were healthy when CDK1 can compensate for their loss by forming active complexes with Cyclin A, B, D, and E, indicating that these CDKs are not essential for completion of the mitotic cell cycle [54]. Other studies demonstrated that *Cdk2* KO in mice leads to postnatal loss of all oocytes, indicating that CDK2 is an important factor for oocyte survival, and likely also oocyte meiosis [55,56]. However, *Cdk1* KO mice, unlike *Cdk2*, suffered early embryonic death, suggesting that CDK1 is the only CDK essential for driving the mitotic cell cycle in mammals [57,58]. Furthermore, *Cdk1* KO, but not *Cdk2*, resulted in female infertility due to a failure in the resumption of meiosis in oocytes [54]. Taken together, our observations and these results suggest that lower *Cdks* mRNA levels induced by TubA may block the resumption of oocyte meiosis.

Very recently, Balmus et al [59] and Liu et al [60] reported that NAT10 acetylates p53 at K120 and stabilizes it by counteracting MDM2 action, and that chemical or genetic targeting of NAT10 decreased genomic instability. In this study, the expression patterns of the NAT10 and Ac-p53 (K120) proteins were changed from the nucleolus to the cytoplasm and/or cell membrane during control oocyte maturation, whereas expression was limited to the nucleus with TubA treatment. Furthermore, *Nat10* mRNA levels were significantly decreased with 20 μM TubA treatment compared to control GV or MII oocytes. A previous report suggested that NAT10 translocates to the cytoplasm or membrane to facilitate α-tubulin acetylation and enhance microtubule stability or activate rRNA transcription by binding and acetylating UBF [61]. Even though a *Nat10* biallelic KO was lethal in mice, a more recent study showed that monoallelic *Nat10* KO mice were subfertile [59]. Taken together, our observations suggest that the NAT10 protein expression pattern is closely linked to Ac-p53, and that the levels and localization of the NAT10 protein is critical for oocyte maturation in the mouse.

In conclusion, knockdown screening techniques using chemical inhibitors are extremely valuable tools. However, this system may also have its limitations such as differences in drug penetration rates into oocytes, oocyte state during culture, and the fraction of unaffected oocytes. More studies are required to prove batch effects caused by knockdown screen techniques to improve the performance of cluster analysis.

## MATERIALS AND METHODS

Unless otherwise stated, chemical and media were obtained from Sigma-Aldrich (St. Louis, MO, USA).

### Mice

Mice were housed in wire cages at 22±1°C under a 12 h light-dark cycle with 70% humidity. All studies were approved by the Animal Care and Use committee of Konkuk University (IACUC approval number: Konkuk university 16122) and were done in accordance with Konkuk University guidelines. B6D2F1 (C57BL/6 x DBA) female mice aged from 6 to 8 weeks were used for collecting oocytes as previously described [15].

### Oocyte collection and TubA treatment

Female mice were injected with 5 IU pregnant mare serum gonadotropin (PMSG) and were sacrificed 48 h after PMSG injection. Germinal-vesicle (GV) oocytes were collected using a mouth pipette. Cumulus cells were dispersed with hyaluronidase (300 IU/mL) for 3-5 min in M16 medium. Denuded oocytes were rinsed thoroughly 5 times in M16 medium and then randomly cultured in M16 medium with or without 10 or 20 μM TubA, after covering with sterile mineral oil, under 5% CO_2_ at 37°C for 12 h. TubA (Catalogue no. 27108) was obtained from BPS Bioscience (San Diego, USA) and dissolved in DMSO. In this study, TubA was used at a final concentration of 10 or 20μM, as previously described [15]. *In vitro*-matured MII oocytes were collected if they had a polar body extrusion, and subjected to further analyses.

### RNA library preparation and sequencing

This experiment was performed by TheragenEtex (www.theragenetex.com) according to their protocol. Libraries from pooled (n=100) oocytes (GV, MII, and 10 or 20 μM TubA-treated) were constructed by TheragenEtex. Briefly, total RNA was extracted from GV, MII, and TubA-treated oocytes using the PicoPure® RNA Isolation Kit (Arcturus, Carlsbad, CA, USA) following the manufacturer’s instructions. Prior to cDNA library construction, 2 μg of total RNA was treated with DNase I, and magnetic beads with Oligo (dT) were used to enrich poly (A) mRNA. The efficacy of each step of library construction was ascertained using a 2100 Bioanalyzer (Agilent Technologies, CA, USA). The library was sequenced using a HiSeq 2500 sequencer (Illumina, San Diego, CA, USA). Cluster generation was performed, followed by 2×100 cycle sequencing reads separated by a paired-end turnaround. Image analysis was performed using the HiSeq control software version 1.8.4.

### Quantification of gene expression and analysis of differentially expressed genes

This experiment was analyzed by LAS (http://www.lascience.co.kr) according to their protocol. To quantify the mapped reads on the reference genome, cufflinks with the strand-specific library option, --library-type=fr-firststrand and other default options were used. Gene annotation of the mm10 reference genome from UCSC genome (https://genome.ucsc.edu) in GTF format was used as a gene model and the expression values were calculated in the Fragments Per Kilobase of transcript per Million fragments mapped (FPKM) unit. Unsupervised clustering by in-house R scripts of the normalized expression values from the few hundred selected differentially expressed genes (DEGs) was used to compare the expression profiles among the samples. The scatter plots for the gene expression values, the volcano plots for the expression-fold changes, and *p*-values between the two selected samples also were drawn by in-house R scripts.

### Functional category analysis

To elucidate the biological functional role of the differential gene expression between the compared biological conditions, we tested gene set overlapping between the analyzed differentially expressed and functionally categorized genes, including biological processes, using Gene Ontology (GO), Kyoto Encyclopedia of Genes and Genomes (KEGG) pathways, and Ingenuity Pathway Analysis (IPA). Network analysis was performed using the IPA web-based application (QIAGEN, Ingenuity Systems). The integrated genome viewer (IGV) was used for genomic annotation as IGV supports various genomic annotation formats [62] and visual representation of annotations follows many of the conventions introduced by the UCSC Genome Browser. Graphs were visualized using GraphPad Prism v6.0 (GraphPad Software, Inc., CA, USA). Venn diagrams were constructed using the web-based program at http://bioinfogp.cnb.csic.es/tools/venny/. RNA sequencing data were deposited in GEO with the accession number GSE115008.

### Immunostaining and confocal imaging

Immunostaining and confocal imaging were prepared as described earlier [15]. Briefly, oocytes were fixed in 4% paraformaldehyde for 30 min and permeabilized for 20 min with PBS containing 0.5% Triton X-100. Permeabilized oocytes were blocked for 2 h at room temperature in 1% bovine serum albumin (BSA) or 10% normal goat serum, then incubated at 4°C overnight with the primary antibodies [anti-CDK1-FITC (ab203852, Abcam), anti-Cyclin B1-Alexa Fluor® 647 (ab215945, Abcam), Anti-NAT10 (ab194297, Abcam), and anti-acetyl-p53 at K120 (ab78316, Abcam)]. All stained samples were mounted using Vectashield mounting medium containing 4′,6-diamidino-2-phenylindole (DAPI) (H-1200, Vector Laboratories, Burlingame, CA, USA). Images were acquired using a confocal laser scanning microscope (LSM800, Zeiss, Germany), and the Fiji open-source platform was used to process and measure image fluorescence intensity, according to a previous report [63].

### Quantitative real time reverse transcription PCR (RT-qPCR)

Total RNAs were extracted from GV, MII, and TubA-treated oocytes using the Dynabeads mRNA Direct Kit (Thermo Fisher Scientific, Rockford, IL, USA) according to the manufacturer’s instructions, respectively. RT-qPCR was performed using a ViiA 7 Real-time PCR system (Applied Biosystems, OR, USA). Target gene expression levels were normalized to *Gapdh* mRNA expression, which was unaffected by TubA treatment. The RT-qPCR primer sets are listed in Table 3. RT-qPCR was performed independently in triplicate for each of the different samples, and the data are presented as the mean values of the gene expression levels measured in the TubA treated samples *versus* the controls, according to a previous report [15].

### Data analysis

All analysis for real time RT-qPCR and RNA-seq are presented as the mean±S.E.M [15]. Each experiment was performed at least three times and subjected to statistical analysis. One-way analysis of variance (ANOVA) was first performed to determine differences among groups (*: p<0.05; **: p<0.01). Fisher’s post hoc test was then performed to determine significant differences between pairs. Values of p<0.05 and p<0.01 were considered significant. Statistical tests were performed using Stat View software version 5.0 (SAS Institute Inc., Cary, NC, USA).

## SUPPLEMENTARY MATERIAL

Supplementary Tables
