# Supplementary material for: Tubastatin A inhibits HDAC and Sirtuin activity rather than being a HDAC6-specific inhibitor in mouse oocytes
Source: Aging (Albany NY). 2019 Mar 26;11(6):1759–77. doi: 10.18632/aging.101867 (PMC6461172; doi:10.18632/aging.101867)
Supplement: Supplementary Tables [file aging-11-101867-s001.pdf]

SUPPLEMENTARY MATERIAL

Supplementary Table 1. Top 10 KEGG pathway analysis up-regulated in TubA/GV pair, compared to MII/GV pair.

Ubiquitin mediated proteolysis	21	Ube3b, Ddb1, Ube2g2, Ube4b, Skp2, Cdc34, Ube3c, Herc1, Cdc26, Birc3, Plas4, Slah1b, Slah1a, Trim32, Uba3, Ddb2, Mdm2, Itch, Rchy1, Ube2s, Cul1
Cell cycle	20	Trp53, Cdc7, E2f4, E2f5, Dbf4, Skp2, Ywhab, Pkmyt1, Chek1, Rb1, Pttg1, Cdc26, Ccnb1 (cyclin B1), Rad21, Eps300, Ccnd3, Mdm2, Ccna2, Cul1, Stag1
Insulin signaling pathway	20	Map2k1, Prkab2, Mknk2, PrkabB1, Raf1, Pde3b, Rhoq, Foxo1, Socs4, Ppp1cb, Akt1, Prkar2b, Ppp1r3d, Nras, Prkar2a, Sorbs1, Rheb, Prkaca, Shc1, Crk
Prostate cancer	15	Trp53, Fgfr2, Map2k1, Pdgfa, Raf1, NfkbIa, Lef1, Foxo1, Nfkb1, Rb1, Pten, Akt1, Nras, Ep300, Mdm2
Pathways in cancer	34	Fgfr2, Pdgfa, Fgf9, Foxo1, Nfkbia, Nfkb1, Cdh1, Gli3, Pten, Akt1, Rac1, Rala, Trp53, Dvl2, Ctbp2, Map2k1, Ralbp1, Runx1t1, Skp2, Raf1, Lef1, Rb1, Ctnna1, Birc3, Dapk2, Nras, Ccdc6, Rassf5, Ep300, Hif1a, Itga6, Pias4, Mdm2, Crk
p53 signaling pathway	12	Ccnb1, Trp53, Ccnd3, Siah1b, Siah1a, Shisa5, Ddb2, Mdm2, Chek1, Rrm2b, Rchy1, Pten
Renal cell carcinoma	12	Akt1, Nras, Ep300, Hif1a, Map2k1, Pdgfa, Pak4, Rac1, Rap1a, Raf1, Flcn, Crk
Tight junction	18	Ppp2r1a, Gnai3, Gnai2, Cldn4, Gnai1, Mpp5, Cask, Ctnna1, Pten, Tjap1, Llgl2, Csnk2a2, Akt1, Nras, Csnk2a1, Ash1l, Pard6g, Mllt4
Progesterone-mediated oocyte maturation	13	Gnai3, Gnai2, Map2k1, Gnai1, Raf1, Pkmyt1, Pde3b, Cdc26, Ccnb1, Akt1, Rps6ka6, Prkaca, Ccna2
MAPK signaling pathway	28	Trp53, Fgfr2, Map2k1, PdgfA, Fgf9, Taok3, Ptprr, Mknk2, Ppm1a, Raf1, Nfkb1, Tab2, Daxx, Atf2, Akt1, Nras, Rps6ka6, Dusp1, Dusp14, Pla2g12a, Rac1, Rap1a, Prkaca, Crk, Dusp7, Rasa1, Rasa2, Ppp5c

Supplementary Table 2. Top 10 KEGG pathway analysis down-regulated in TubA/GV pair compared to MII/GV pair.

Ribosome		58	Rpl18, Rpl36a, Rpl19, Rpl14, Gm6251, Rpl22l1, Rps3, Rpl39l, Fau, Rpl10, Rpl12, Rps27a, Rpl36al, Rpl35a, Rps4x, Rps18, Rps19, Rps17, Rps15, Rps13, Rps10, Ubb, Kpna2, Gm12191, Rpl27a, Rpl35, Rpl36, Rpl38, Rpl39, Rps25, Rps26, Mrpl13, Rpl30, Rpl7, Rps29, Rpl6, Rpl31, Rpl8, Rpl3, Gm15772, Rpl5, Rpl10a, Rpl7a, Rps20, Rpl4, Rps21, Rps23, Rps24, Rpsa, Rpl26, Rps9, Rpl27, Rpl24, Rpl23A, Rps6, Rps5, Rpl28, Rps7, Rpl29, Rpl18a, Rpl23, Rpl13a, Rpl21
Parkinson's disease		53	Atp5d, Atp5e, Uqcrc1, Cyc1, Uchl1, Uqcrfs1, Uqcrq, Cox5b, Ndufs7, Ndufs6, Ndufs5, Ndufs8, Atp5o, Ndufs3, Atp5h, Ndufs2, Rps27a, Atp5j, Ndufb10, Cyct, Cycs, Ndufc2, Ube2j1, Gm5801, Cox4I1, Ndufc1, Uqcrh, Ubb, Uqcrb, Ndufb3, Ndufb4, Ndufb5, Ndufb7, Cox7b, Cox7c, Atp5g2, Cox7a2l, Atp5g1, Atp5g3, Ndufa2, Ndufa8, Ndufa9, Ndufa7, Atp5f1, Vdac2, Vdac3, Park7, Ndufv3, Sdhb, Ndufv1, Sdhd, Ndufv2, Cox6a2, Atp5a1
Oxidative phosphorylation		52	Atp5d, Atp5e, Uqcrc1, Cyc1, Uqcrfs1, Uqcrq, Cox5b, Ndufs7, Ndufs6, Ndufs5, Ndufs8, Atp5O, Atp6v0d1, Ndufs3, Cox17, Atp5h, Ndufs2, Atp5k, Atp5j, Ndufb10, Ndufc2, Cox4i1, Ndufc1, Ndufa11, Uqcrh, Uqcrb, Ndufb3, Ndufb4, Ndufb5, Ndufb7, Cox7b, Cox7c, Atp5g2, Cox7a2l, Atp5g1, Atp5g3, Atp6v0c, Atp5j2, Ndufa2, Ndufa8, Ndufa9, Ndufa7, Atp5f1, Lhpp, Ndufv3, Sdhb, Ndufv1, Atp6v1e1, Sdhd, Ndufv2, Cox6a2, Atp5a1
Huntington's disease		58	Atp5d, Atp5e, Clta, Uqcrc1, Cltb, Cyc1, Uqcrfs1, Uqcrq, Cox5b, Ndufs7, Ndufs6, Ndufs5, Ndufs8, Atp5o, Ndufs3, Atp5h, Ndufs2, Atp5j, Ndufb10, Cyct, Cycs, Ndufc2, Cox4i1, Ndufc1, Dctn2, Uqcrh, Uqcrb, Ndufb3, Ndufb4, Ndufb5, Polr2e, Ndufb7, Polr2l, Polr2k, Cox7b, Polr2i, Cox7c, Atp5g2, Cox7a2l, Atp5g1, Atp5g3, Ndufa2, Creb3, Ndufa8, Ndufa9, Ndufa7, Atp5f1, Sod1, Vdac2, Vdac3, Ndufv3, Sdhb, Ndufv1, Bax, Ndufv2, Sdhd, Cox6a2, Atp5a1
Alzheimer's disease		54	Atp5d, Atp5e, Uqcrc1, Cyc1, Uqcrfs1, Uqcrd, Cox5b, Ndufs7, NdufS6, Ndufs5, Ndufs8, Atp5o, Ndufs3, Atp5h, Ndufs2, Atp5j, Ndufb10, Cyct, Cycs, Ndufc2, Cox4i1, Ndufc1, Cdk5, Psen1, Uqcrh, Bace2, Uqcrb, NdufB3, Hsd17b10, Ndufb4, Ndufb5, Ndufb7, Aph1c, Cox7b, Cox7c, Atp5g2, Cox7a2l, Atp5g1, Atp5g3, Ppp3cc, Ndufa2, Ndufa8, Ndufa9, Ndufa7, Atp5f1, Bad, Ndufv3, Sdhb, Ndufv1, Sdhd, Ndufv2, Cox6a2, Calm3, Atp5a1, Calm2
Proteasome ubiquitin mediated proteolysis		19	Psmb5, Psmf1, Psmb4, Psmd14, Psmb7, Psmc5, Psmd13, Psmb6, Psma6, Psmc4, Psmb1, Psme2, Psmc3, Psmc2, Psmc1, Psma3, Psmd4, Psmd6, Psmd8
Pyrimidine metabolism		27	Polr2e, Polr2l, Polr2k, Polr2i, Dtymk, Upp1, Znrd1, Tk2, Tyms, Nt5m, Nt5c3, Uck2, Entpd1, Nudt2, Polr3h, Polr3k, Polr1e, Polr1d, Polr1c, Polr3a, Polr3c, Nme6, Pold4, Umps, Nme3, Nnm1, Pold2

RNA polymerase		12	Polr3h, Polr2e, Polr3k, Polr1e, Polr2l, Polr1d, Polr2k, Polr2i, Znrd1, Polr3a, Polr1c, Polr3c
Pentose phosphate pathway		10	Aldoa, Pgm2, Pgls, G6pdx, Fbp1, Pfkp, Dera, Tkt, Fbp2, Gpi1
DNA replication		11	Rpa2, Pold4, Rfc3, Rfc4, Ssbp1, Rfc2, Pold2, Rnaseh2a, Fen1, Rnaseh2c, Mcm5

Supplementary Table 3. List of primers used in this study.

Genes	Forward	Reverse
Cdc14b	GTGAAGAAGAGCCGCAG	GCTGTAGAGAATGGCAAAAC
Arp3	CGCCATGGTATAGTTGAAGA	AGTGGAGGTTCAGTCAAAAG
Aurora c	CGTACAGCCACGATAATACA	CCTGTGAATCACCTTCTTCT
Cops3	TGTGGAAAGAAAACAGCCC	GTCAGTTGGTTGGTATTCATC
Cops5	AAACCCTGGACTAAGGATCA	TCACCATTTTCAGTAGAGCC
Tkt	ATGGCATACACAGGCAAATA	TTGTAAATTCCAGCAAAGGC
Obox1	GGCACTATCAGTTGGTGTTA	ATATTCTGGAGGTTCATCCG
Gapdh	AGGTCGGTGTGAACGGATTTG	TGTAGACCATGTAGTTGAGGTCA
